# Glucagon-like peptide-1 receptor agonists and suicide risk in individuals with diabetes and Cannabis use disorder

**DOI:** 10.1016/j.pmedr.2025.103244

**Published:** 2025-09-12

**Authors:** Yesh Dhruva, Erick Messias, Ping-I Lin

**Affiliations:** a Department of Psychiatry and Behavioral Neuroscience, Saint Louis University School of Medicine, St. Louis, MO, USA; b Discipline of Psychiatry and Mental Health, School of Clinical Medicine, University of New South Wales, Sydney, NSW, Australia

**Keywords:** GLP-1 receptor agonists, Suicide risk, Cannabis use disorder, Type 2 diabetes mellitus, Survival analysis

## Abstract

**Objective:**

The current study is to examine the association between GLP-1 receptor agonist (GLP-1 RA) use and suicide attempt risk in individuals with type 2 diabetes mellitus (T2DM), including those with comorbid cannabis use disorder (CUD).

**Methods:**

We conducted a retrospective study using the TriNetX Research Network. Adults aged 30–85 years with a diagnosis of T2DM were categorized by GLP-1 RA exposure and CUD sourced from over 20 countries across the Americas, Europe-Middle East-Africa, and Asia-Pacific from 2003 to 2023. Individuals with a prior diagnosis of suicidal ideation were excluded. The primary outcome was a new diagnosis of suicide attempt within one year. Cox proportional hazards models were used to estimate adjusted hazard ratios (aHRs), controlling for age, sex, depression, body mass index, and glycated hemoglobin A1c (HbA1c) levels. Kaplan-Meier curves were used to visualize cumulative risk.

**Results:**

Among 6,424,228 individuals with T2DM, GLP-1 RA use was associated with significantly lower risk of suicide attempt compared to unexposed individuals (aHR: 0.63; 95 % CI: 0.47,0.85). In contrast, patients with CUD exhibited markedly elevated risk (aHR: 5.50; 95 % CI: 4.39,6.89). This elevated risk persisted among those concurrently using GLP-1 RAs and diagnosed with CUD (aHR: 5.75; 95 % CI: 3.42,9.69). No significant risk reduction was observed among CUD patients using GLP-1 RAs compared to those not using them (aHR: 1.00; 95 % CI 0.37–2.69).

**Conclusions:**

The inverse association between GLP-1 RAs and suicide attempt was attenuated among cannabis users. These findings underscore the importance of addressing substance use when assessing psychiatric risk in diabetes care.

## 1 Introduction

An increasing number of individuals with type 2 diabetes mellitus (T2DM) have become cannabis users. A recent national study reported a 34 % rise in past-month cannabis use among U.S. adults with diabetes between 2021 and 2022 (Han et al., 2024). Emerging evidence indicates that individuals with T2DM also face an elevated risk of suicide compared to the general population, with studies suggesting nearly a twofold increase in suicide-related mortality and self-harm behaviors (Fan et al., 2024), although cannabis use has been found to be associated with lower risk of insulin resistance (Ngueta and Ndjaboue, 2020; Penner et al., 2013). The joint effects of T2DM and cannabis use on mental health conditions such as suicidal behaviors are therefore paradoxical and warrant further investigation.

Mental health conditions for diabetic individuals are likely driven by a complex interplay of factors, including the psychological burden of managing a chronic illness, the frequent presence of comorbid depression and anxiety, and the neurobiological effects of dysregulated glucose metabolism (Moulton et al., 2015). The daily demands of diabetes self-care and fear of complications can contribute to persistent distress and feelings of hopelessness. Furthermore, depression and diabetes often co-occur and share genetic, lifestyle, and psychosocial risk factors (Fanelli et al., 2025), which may account for co-occurrence of T2DM and suicidal behaviors.

Despite this concern about mental health crises, suicide prevention strategies rarely target diabetic populations. Further, little is known about how specific diabetes treatments may influence psychiatric outcomes. A growing body of evidence suggests that some diabetes medications may have beneficial effects on mental health. For instance, certain treatments such as glucagon-like peptide-1 receptor agonists (GLP-1 RA) and metformin have shown promise in reducing neuroinflammation and improving mood symptoms (Chen et al., 2024; Yang et al., 2024). GLP-1 RAs, originally developed for glycemic control and weight loss, have attracted growing interest for their potential neuropsychiatric benefits (Fortin et al., 2020a, Fortin et al., 2020b). However, their role in suicide prevention remains unclear, with mixed findings across studies (Bezin et al., 2025; Ueda et al., 2024). Understanding whether optimizing medical management of diabetes can also reduce suicide risk is, hence, a critical area of research.

Given the rising rates of cannabis use among diabetic patients — particularly in states with expanding legalization, there is an urgent need to evaluate the relationship between GLP-1 RA use and suicide risk among individuals with T2DM and cannabis use disorder (CUD). We have leveraging the TriNetX research network, which provides access to de-identified electronic health records from over 100 million patients across diverse geographic and sociodemographic backgrounds in the U.S., to conduct robust, population-scale analyses. This approach enables us to capture real-world variation in treatment patterns and outcomes, and to apply advanced analytic techniques that account for confounding and effect modification.

## 2 Methods

We conducted a retrospective cohort study using the TriNetX Research Network, a federated health research database comprising de-identified electronic health records from over 100 million patients. The study population included adults aged 30 to 85 years with a diagnosis of type 2 diabetes mellitus (T2DM), defined by ICD-10 code E11. The index event was determined as the first recorded T2DM diagnosis within the preceding 20 years (2003−2023), ensuring uniform follow-up availability and reducing potential survival bias by anchoring the start of observation at initial diagnosis.

We defined four mutually exclusive cohorts based on the presence or absence of: (1) active GLP-1 RA prescriptions (Anatomical Therapeutic Chemical code A10BJ), and (2) cannabis-related disorder diagnoses (ICD-10 code F12), recorded any time prior to or concurrent with T2DM diagnosis. Patients were required to have at least one year of follow-up and no history of suicidal ideation (ICD-10: R45.851) on or before the index date to minimize confounding from preexisting suicidality.

The primary outcome was a new diagnosis of suicide attempt (ICD-10: T14.91) within 365 days of the index event. Covariates included age, sex, body mass index percentile, HbA1c levels, and a history of depressive disorder (ICD-10: F32/F33) as a proxy for baseline psychiatric risk. We adjusted for these covariates to account for demographic, metabolic, and mental health-related confounding.

We used Cox proportional hazards models to estimate hazard ratios (HRs) for suicide attempt across exposure groups, using individuals without GLP-1 use or cannabis use as the reference. Kaplan-Meier survival curves were generated to visualize cumulative incidence over time. To mitigate immortal time bias, all exposure and covariate data were assessed and fixed at baseline (the index event date), and follow-up was limited to 365 days post-index for all individuals. We used the built-in Advanced Analytics suite, which incorporates workflows in Python, R, SQL, and Scala, to perform real-time cohort comparisons and survival analyses within the TriNetX platform. This study used only de-identified aggregate data from the TriNetX Research Network and, in accordance with institutional policy, was not considered human subject research; therefore, ethics approval was not required.

## 3 Results

**Fig. 1:**
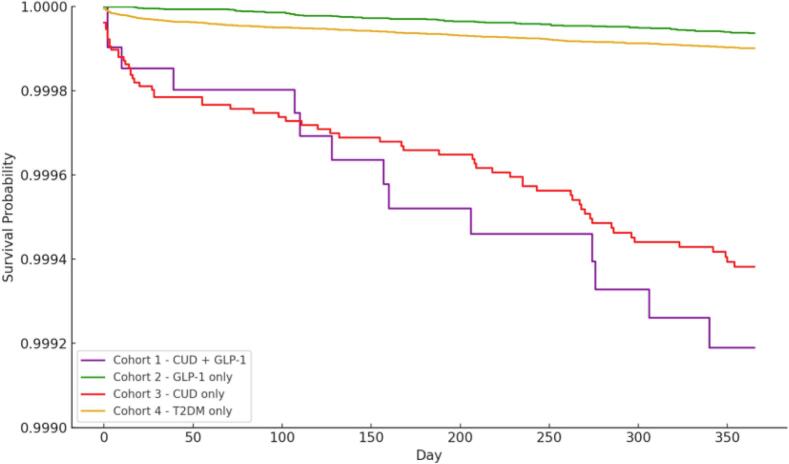
Kaplan–Meier curves of suicide attempt risk in adults with type 2 diabetes by glucagon-like peptide-1 receptor agonist therapy and cannabis use disorder status, Global, 2003–2023.

The data on a total of 6,424,228 patients with T2DM were extracted to construct four cohorts based on GLP-1 RA use and CUD status. In adjusted Cox proportional hazards models (Table 1), individuals prescribed GLP-1 RA had a significantly lower risk of suicide attempt compared with controls (hazard ratio [HR], 0.63; 95 % CI, 0.47,0.85). In contrast, diabetic patients with CUD alone had a markedly increased risk relative to T2DM controls (HR, 5.50; 95 % CI, 4.39,6.89), similar to those with both CUD and GLP-1 RA use (HR, 5.75; 95 % CI, 3.42,9.69). There was no significant difference in risk of suicide attempt when comparing individuals with CUD prescribed with GLP-1 RAs versus those with CUD alone (HR, 1.00; 95 % CI, 0.37,2.69). The Kaplan-Meier curves for these four cohorts are illustrated in Fig. 1.

**Table 1:** Survival analysis of suicide attempt risk among adults with type 2 diabetes receiving glucagon-like peptide-1 receptor agonist therapy with or without cannabis use disorder, Global, 2003–2023.

Comparison Group	Reference Group	Cohort 1 (N)	Suicide Attempts (N)	Cohort 2 (N)	Suicide Attempts (N)	Hazard Ratio (95 % CI)
GLP-1 only	Control	1,042,181	56	6,424,228	497	0.63⁎ (0.47, 0.85)
CUD only	Control	131,154	64	6,424,228	497	5.50⁎ (4.39, 6.89)
CUD + GLP-1	Control	20,974	14	6,424,228	497	5.75⁎ (3.42, 9.69)
CUD + GLP-1	CUD Only	20,403	14	128,729	64	0.999 (0.37, 2.69)

GLP-1 indicates the treatment with Glucagon-like Peptide-1 Receptor Agonists; CUD refers to cannabis use disorder; T2DM refers to type-2 diabetes mellitus. N indicates the sample size. All models adjusted for age, sex, body mass index, glycated hemoglobin A1C test levels, and depressive disorders.

⁎ *p*-value <0.05.

## 4 Discussion

Patients with T2DM who were treated with GLP-1 RAs had the lowest observed risk of suicide attempt, suggesting a potential protective effect. However, this association did not persist among individuals with comorbid CUD, indicating that CUD may attenuate or override the neuropsychiatric benefits of GLP-1 RAs. These findings highlight the importance of accounting for comorbid substance use when evaluating psychiatric outcomes and developing suicide prevention strategies in T2DM populations. Emerging evidence suggests neuroprotective effects of GLP-1 RAs, potentially modulating brain reward pathways involved in addiction and mood regulation, which could potentially confer protection against altered mental behaviors (Erbil et al., 2019; Fortin et al., 2020a).Thus, caution is warranted in interpreting these findings. A recent review of the literature examining the relationship between suicide and GLP-1 RAs found no consistent evidence of increased suicide risk in GLP-1 RA users, reinforcing the need for real-world data to inform future prospective studies. (Di Stefano et al., 2025).

Several limitations should be considered. First, this study relied on diagnostic codes from electronic health records, which may underreport or misclassify cannabis use, GLP-1 RA exposure, and suicide-related outcomes due to incomplete documentation or variability in coding practices across institutions. Given underreporting of substance use in electronic health records, we used a single ICD-10 F12 code to define individuals with CUD in order to capture the largest possible cohort. This approach may have led to misclassification bias and attenuated associations. Second, residual confounding also remains, as anxiety, psychosis, personality disorders, and psychotropic medications were not uniformly captured. Some other clinical variables such as diabetes duration and severity, glycemic control, psychiatric comorbidities (e.g., depression, anxiety), social determinants of health, and use of other substances (e.g., alcohol, tobacco, illicit drugs) were not fully captured or controlled for, potentially biasing results. The reliance on electronic health record data also precludes assessment of patient-reported outcomes, symptom severity, or psychosocial stressors, which are relevant to suicide risk but rarely documented in structured data fields. Additionally, medication exposure was modeled as a binary and time-invariant variable, which does not reflect differences in treatment duration, dose, changes in therapy, or adherence —factors that may influence both psychiatric outcomes and suicide risk. Current TriNetX access did not provide longitudinal data on prescription duration, dosage, or treatment changes. We therefore chose a binary exposure definition to maximize cohort size and minimize misclassification. Finally, as an observational study, causal inference is inherently limited and associations observed cannot establish directionality or causality. The generalizability of our findings may also be restricted to populations represented within the TriNetX network, which may not reflect broader or more diverse patient groups. Despite these limitations, our study provides valuable insights into the interplay between pharmacotherapy, substance use, and suicide risk in individuals with T2DM, highlighting areas for future research and clinical attention.

These results have important clinical implications, particularly for the understudied population of diabetic cannabis users. Given the high prevalence of both T2DM and CUD, clinicians should be vigilant in screening for substance use when prescribing GLP-1 RAs and assessing suicide risk. The possible attenuation of GLP-1 RAs' protective neuropsychiatric effects by CUD suggests that standard diabetes management strategies may not confer the same mental health benefits in this subgroup. This highlights the need for integrated care approaches that address both metabolic and psychiatric needs, including targeted interventions for substance use and enhanced mental health monitoring. Moreover, the null association observed in the CUD + GLP-1 RA subgroup (HR = 1.00) is noteworthy. Several factors may explain this finding. Biologically, cannabis use has been linked to alterations in dopaminergic and endocannabinoid signaling that could counteract the neuroprotective or mood-stabilizing effects of GLP-1 receptor agonists. Clinically, individuals with comorbid CUD often present with greater psychiatric burden, such as including higher rates of polysubstance use, medication non-adherence, and social stressors, which may overshadow any modest protective effects of GLP-1 therapy on suicidality.

In conclusion, these findings call for the development of clinical guidelines that explicitly consider comorbid substance use in diabetes care, as well as further research to elucidate the neurobiological mechanisms underlying the interaction between GLP-1 RAs and cannabis on mood and suicidality. Prospective studies with more granular data on substance use patterns, medication adherence, and psychiatric outcomes are warranted to inform personalized treatment strategies. Ultimately, a multidisciplinary approach that incorporates endocrinology, psychiatry, and addiction medicine may be essential to optimize both physical and mental health outcomes in this vulnerable population (Eren-Yazicioglu et al., 2021).

GLP-1 refers to treatment with Glucagon-like Peptide-1 Receptor Agonists; CUD indicates the diagnosis of cannabis use disorder; T2DM refers to type-2 diabetes mellitus. Survival probability refers the proportion of individuals who have not been found to exhibit suicide attempts.

## Disclosure of Ethical Compliance

TriNetX is a federated, anonymized databased, and hence the current study is not considered human subject research, which does not require ethics approval.

## CRediT authorship contribution statement

**Yesh Dhruva:** Writing – review & editing, Writing – original draft, Formal analysis, Data curation. **Erick Messias:** Writing – review & editing, Writing – original draft, Supervision, Conceptualization. **Ping-I Lin:** Writing – review & editing, Supervision, Investigation, Conceptualization.

## Sources of Funding

No sources of funding.

## Declaration of competing interest

The authors declare that they have no known competing financial interests or personal relationships that could have appeared to influence the work reported in this paper.
